# Optimal release timing of temporary drain clamping after total knee arthroplasty

**DOI:** 10.1186/s13018-017-0550-y

**Published:** 2017-03-21

**Authors:** Yoon Sang Jeon, Jun Sung Park, Myung Ku Kim

**Affiliations:** Department of Orthopedic Surgery, College of Medicine, Inha University Hospital, 7-206, 3-Ga Sinheung-dong, Jung-gu, Incheon 400-711 South Korea

**Keywords:** Total knee replacement, Bleeding, Clamping

## Abstract

**Background:**

Bleeding control is critical after total knee arthroplasty (TKA). The purpose of this study was to evaluate the optimal time to release the clamped drain after TKA.

**Methods:**

We performed unilateral TKA in 120 patients using three methods of drainage. Group A (*N* = 40) had a 3-hour clamp applied, and group B (*N* = 40) had a 4-hour clamp applied. Group C (*N* = 40) underwent conventional negative drainage. We evaluated the drainage volume, as well as the hemodynamic markers, transfusion volume, visual analog scale (VAS) scores, and range of motion (ROM).

**Results:**

The drained blood volume in groups A and B was significantly less than that in group C. No significant difference was found between groups A and B. The level of hemoglobin in group A was significantly higher than that in group C at 2 days after surgery. The ROM of groups A and C was larger than that of group B at 5 days after surgery. Furthermore, the VAS scores of groups A and C were significantly lower than those of group B at both 2 and 5 days after surgery.

**Conclusions:**

The temporary drain clamping method after TKA significantly reduced the volume of bleeding and blood transfusion. The 3-h clamping method reduced the drained volume as effectively as the 4-hour clamping method and resulted in less acute phase pain and more rapid recovery of ROM than the 4-hour clamping method. In conclusion, we recommend 3-h clamping after TKA as the optimal release time to reduce blood loss and acute phase pain.

## Background

Total knee arthroplasty (TKA) presents a high risk of significant bleeding and blood transfusion. It is costly, it carries a high risk of disease transmission, and it warrants extended hospitalization [1, 2]. To reduce and control the bleeding, various methods have been suggested, including tranexamic acid [3, 4], temporary drain clamping [5–7], and the use of a fibrin agent [8]. Controversy concerning the preferred drainage method after TKA persists. Although drainage helps prevent hematoma and is effective in reducing pain and edema [9–11], it carries a risk of increasing bleeding when removing the tamponade and thus providing a subsequent route of infection. Temporary drain clamping is one of several methods devised to reduce bleeding after surgery. The drain is clamped for several hours after surgery to form a tamponade before opening. This method is considered effective in reducing bleeding [12]. Prior studies have examined many methods, such as the intermittent method [13–15] and the specific hour-drain clamping after surgery (e.g., 1 [16], 2 [17], 4 [18], 12 [19], and 24 h [20]). However, the most suitable method has not yet been established.

Previous studies have not compared various clamping methods to determine the most effective method at a single research center. Generally, bleeding after TKA is known to be concentrated immediately after the surgery (37% in 2 h, 49% in 3 h, and 55% in 4 h) [21]. During this period, temporary clamping of the drainage to form a tamponade may reduce blood loss. However, if the drainage has been clamped for too long, bleeding that occurs after the formation of a tamponade cannot be removed, thus, causing the wound to form a hematoma [7, 22]. The most critical time period for forming the tamponade is assumed to be 3–4 h after the operation, when most bleeding occurs. The purpose of this study is to evaluate the optimal time to release the clamped drain after TKA.

## Methods

From February 2014 to December 2015, 143 unilateral TKAs were performed at our hospital. The inclusion criterion for this study was primary TKA using the same prosthesis: Nexgen-LPS Flex^Ⓡ^ (Zimmer, Warsaw). Twenty-three patients were excluded from the study including 10 cases of revised TKA, 7 cases in which a wedge or block was used due to a bony defect, and 6 cases in which different prostheses were used (LCCK, Zimmer, Warsaw) due to medial or lateral instability. We retrospectively analyzed the data prospectively collected from 120 patients who underwent primary TKA due to degenerative osteoarthritis. None of the patients had a bleeding disorder. The protocol of this study was approved by the Inha University Hospital Institutional Review Board (approval number: IUH-IRB 15–1637). All subjects were randomly assigned to three groups using concealed envelopes. The 3-h drain clamping method was used for 40 patients (group A), and 4-h drain clamping was performed on 40 patients (group B). Clamping was not performed on 40 patients, but conventional negative suction drainage (group C) was performed. Drain clamping was performed after closure of the joint capsule and the pressure reduction of the tourniquet. A BAROVAC (400 mL, negative pressure-90 mmHg, Sewoon Medical) was used as the hemovac, and clamping was performed by the anti-reflux valve. Then, clamping was released after the specified time based on random allocation. If the drainage volume was less than 30 cc, the drains were removed in all cases.

All operations were performed by one senior surgeon (MKK), who is experienced in TKAs, and the same surgical techniques were used for all patients. Under spinal anesthesia, a pneumatic tourniquet was applied with a pressure of 350 mmHg using a midline skin incision, and a medial parapatellar approach. Additionally, we performed a partial synovectomy for all cases. Regarding the implant, a posterior cruciate ligament replacement system, Nexgen-LPS Flex^Ⓡ^ (Zimmer, Warsaw), was used with cement fixation. A femoral intramedullary alignment rod was used in all cases, and the femoral canal was blocked with a bone plug before implantation of the femoral prosthesis. A 3.2-mm drainage tube was inserted into the medial gutter of the joint. The synovium was sutured with a simple running suture with 3–0 vicryl, and the joint capsule and tendons were sutured with simple interrupted sutures with 1–0 vicryl. The subcutaneous tissue were sutured with simple buried sutures with 2–0 vicryl, and the skin was sutured with surgical skin staples. After the skin was sutured, a standard aseptic compression dressing was applied.

To reduce the influence of drugs on bleeding, all anti-platelet agents such as aspirin were stopped 1 week before the operation and restarted two weeks after the operation [23]. To reduce intraoperative bleeding, all patients were administered 1 Klobusitzky Unit (KU) of hemocoagulase intravenously before the operation. To prevent deep vein thrombosis (DVT) or pulmonary thromboembolism (PTE), all patients wore anti-embolism stockings and intermittent pneumatic compression devices (IPC) for 2 weeks after the operation. In addition, after removal of the drainage, all patients were given 10 mg of Xarelto® (rivaroxaban) daily for 2 weeks to prevent DVT or PTE. Rivaroxaban (Xarelto®) is an oral direct factor Xa inhibitor. Factor Xa is activated via the extrinsic and intrinsic pathways and is the rate-limiting step in the propagation of thrombin generation. The direct Xa inhibitor rivaroxaban (Xarelto®) prevents venous thromboembolic events after major elective orthopedic surgery [24].

Preoperative laboratory examinations, such as hemoglobin (Hb) and hematocrit (Hct), measurements were performed on all patients. In terms of blood transfusions, one unit of RBCs was transfused to the patients if their Hb value was less than 9 g/dL on the postoperative day (POD) or if dizziness and compromised clinical criteria persisted (such as tachycardia and hypotension). If Hb levels decreased to less than 8 g/dL, the patients received two units of RBCs [22, 25].

Perioperative blood loss was not measured. However, the Hb values before the operation and the postoperative Hb were measured to calculate the estimated blood loss (EBL) using Good and Nadler’s formula [26]. Hidden blood loss (HBL) was calculated by subtracting the amount of drainage blood from the EBL [27].

This study evaluated the VAS scores at POD 2 and 5 and complications such as symptomatic DVT and PTE, which may occur during clamping of the drainage system. Continuous passive motion (CPM) was performed for all patients after Hemovac® removal, while the angles of CPM were recorded daily. Surgical wounds were evaluated on the second POD and included major bruising, blisters, oozing, and subcutaneous hematoma. Major bruising was classified as bruising of more than 5 cm around the wound, and oozing was defined when the wound required reinforcement of the dressing. In addition, superficial or deep wound infection was assessed until the day of discharge.

Data are shown as the means ± standard deviation (S.D.) for normally distributed data and as medians (ranges) for non-normally distributed data. The measured data of the three groups were analyzed using one-way ANOVA for normally distributed data, and the Kruskal-Wallis test was used to analyze non-normally distributed data. Fisher’s exact test was used to compare the differences in complications. In cases with *P* < 0.05, the results were considered statistically insignificant. The statistical program SPSS version 20.0 (SPSS, Chicago, Illinois) was used.

## Results

The demographic data of patients are shown in Table 1. No significant difference was observed in the sex ratio, age, BMI, preoperative Hb, and ROM among the three groups.

**Table 1:** Demographic data of each group

Characteristic	Group A (*n* = 40)	Group B (*n* = 40)	Group C (*n* = 40)
Sex (female:male)	35:5	31:9	32:8
Age	70.3 ± 8.5	71.3 ± 7.8	67.2 ± 10.2
BMI	26.3 ± 3.7	25.9 ± 2.8	26.9 ± 4.9
PreOP ROM (degree)	107.1 ± 9.1	108.4 ± 12.6	107.6 ± 11.5
PreOP Hb (g/dL)	12.7 ± 1.8	12.2 ± 1.8	12.5 ± 1.4

*Abbreviations*: *BMI* body mass index, *PreOPHb* preoperative hemoglobin, *Preop ROM* preoperative range of motion

Group A:3-h clamp, group B:4-h clamp, group C:non-clamp

Values were presented as mean ± SD

The mean Hb value on POD 2 in the non-clamping group was lower than that in the other groups (*P* = 0.03) (Table 2). The EBL of group C was higher than that of the other groups (*P* = 0.011). The mean drainage volume of group C was significantly higher than that of the other groups (*P* = 0.01). No significant differences in the volume of HBL were observed among the three groups (*P* = 0.145). The transfusion volume in group C was higher than that in the other groups (*P* = 0.004). All drains were removed within 5 days. The mean removal times were 3.58 (2–5) days for group A, 3.45 (2–5) days for group B, and 3.63 (2–5) days for group C (*P* = 0.711).

**Table 2:** Blood loss, transfusion requirement, and wound complications

	Group A (*n* = 40) (3-h clamp)	Group B (*n* = 40) (4-h clamp)	Group C(*n* = 40) (non-clamp)	*P* value
Hb (g/dl)				
Day 2	9.9 ± 1.8	9.6 ± 1.5	9.0 ± 1.3	0.030
Day 5	10.2 ± 1.2	10.1 ± 1.0	9.6 ± 1.2	0.067
Estimated blood loss (ml)	1050.5 ± 450.6	1078.1 ± 633.9	1391 ± 554.3	0.011
Drained blood volume (ml)	545.1 ± 186.5	535.5 ± 166.1	652.6 ± 211.0	0.010
Hidden blood loss	505.3 ± 469.9	573.1 ± 690.4	739.2 ± 557.0	0.145
RBC transfusion (unit)	0.9 ± 0.8	1.0 ± 0.7	1.5 ± 1.0	0.004
ROM at post OP 5 day (degree)	92.5 ± 7.8	84.1 ± 10.6	90.8 ± 9.8	<0.001
VAS				
Day 2^a^	5 (3–9)	6 (3–9)	5 (3–9)	0.022
Day 5^a^	4 (2–9)	5 (3–9)	4 (2–6)	0.009
Wound complications				
Major bruising (*n*)	2	3	2	0.860
Blister (*n*)	1	2	1	0.774
Oozing (*n*)	2	4	1	0.349
Superficial infection (*n*)	0	1	0	0.368

*PostOP Hb* postoperative hemoglobin, *Hb* hemoglobin, *Hct* hematocrit, *POD* postoperative day, *ROM* range of motion

Normally distributed data given as mean ± SD

^a^Median and range

Regarding the degrees of CPM, the mean flexion angle of the ROM for group A was 92.5°, while those of groups B and C were 84.1° and 90.8°, respectively, on POD 5. Statistically, the angles were significantly higher for groups A and C (*P* < 0.001). The VAS scores on POD 2 and 5 were significantly higher in group B (*P* = 0.022 and *P* = 0.09).

No significant differences in complications were noted among the three groups. No symptomatic DVT and PTE occurred in any patient. The mean time of discharge in group A was 12.9 days after surgery, and those of groups B and C were 13.5 and 13.3 days, respectively (*P* = 0.214).

## Discussion

In the present study, the volume of drainage in the clamping groups (A and B) was significantly less than that in the non-clamping group (C). Additionally, no significant difference was observed between the 3 and 4-h clamping groups. In terms of early ROM, the angles of the 4-h clamping group were significantly lower than those of the other groups, and in terms of postoperative pain, the VAS scores of 4-h clamping group were significantly higher than those of the other groups.

Reduction of bleeding and transfusion is a very important consideration in TKA. The temporary drainage clamping method does not require special skills, medication, or costs to control bleeding [5, 6, 15]. The intermittent clamping method was initially introduced in 1984, when it was observed that intermittent clamping was associated with less blood loss than conventional drainage [28]. However, no consensus has been reached regarding the optimal timing of clamping after TKA [22, 29]. If clamping is applied for too long, complications such as hematoma can occur, which can be a source of infection. However, when clamping is removed too soon, the reduction of blood is not effective. Kiely et al. [17] reported that drain clamping for 2 h after a TKA is not effective for reducing the volume of bleeding. In addition, Aksoy et al. [30] reported no difference in blood loss between the 2-h clamping group and the non-clamping group. In meta-analysis studies, the 2-, 1-, and half-hour clamping methods showed no reduction in true blood loss [14, 30]. Accordingly, a longer time is required for drain clamping to form an effective tamponade. Tai TW et al. [29] suggested that the 4-h drain clamping method was statistically valid and could significantly reduce the volume of bleeding. Senthil KG et al. [21] reported that bleeding after a TKA is known to be concentrated immediately after the surgery. The most critical time zone for forming the tamponade is assumed to be 3–4 h after the operation, when most bleeding occurs. In the present study, patients in the 3- and 4-h clamping groups had less drainage volume than those without clamping. These results support prior studies that have investigated drain clamping.

Jung et al. [25] found that even when a difference in the volume of blood drainage occurred, no significant difference in the total blood loss was observed due to HBL. However, in the present study, no significant difference in HBL was observed among all groups. Thus, we concluded that the reduction of the drained volume leads to a reduction of total blood loss. Consequently, we suggest that the clamping method after TKA is effective to reduce total blood loss. According to our results, the drained blood volume and RBC transfusions after TKA in the 3-h clamping group were not significantly different from those of the 4-h clamping group. Therefore, we concluded that the 3-h clamping method reduces the total blood loss as effectively as the 4-h clamping method.

Significantly greater pain was felt by patients in group B on POD 2 and 5. Statistically, the ROM on POD 5 was significantly lower for patients in group B. Theoretically, closed negative pressure drainage can effectively prevent hematoma formation, which is believed to restore early knee function by reducing postoperative swelling [31, 32]. However, clamping of the drainage provides a temporary tamponade effect to reduce blood loss, and delayed release prevents hematoma formation [29]. However, if clamping is applied for too long, bleeding cannot be removed after tamponade formation. This may indicate that clamping can cause blood accumulation, which leads to hematoma formation and pain. When the pressure of the hematoma increases, the accumulated blood can infiltrate into the tissues surrounding the knee joint. Edema formation in the periphery of the knee joint induces pain. Furthermore, Hoffman et al. [33] proposed that joint effusion is a major cause of pain after TKA. Therefore, we speculated that edema was reduced in the 3-h clamping group by removing the hematoma at the appropriate time, resulting in pain relief, and an early ROM relative to the 4-h clamping group.

This study has limitations. First, a relatively small number of cases were included in each group because all the operations were performed by one surgeon in a single center. Thus, a consecutive study is required. Second, although several factors affect blood loss after TKA, such as soft tissue release or insert size, these effects were not evaluated in the present study.

## Conclusions

Use of the temporary drain clamping method after TKA significantly reduced the volume of bleeding and blood transfusion. The 3-h clamping method reduced the drained volume as effectively as the 4-h clamping method and resulted in less acute phase pain and a more rapid recovery of ROM than 4-h clamping. In conclusion, we suggest 3-h clamping after TKA as an optimal time of release for reducing blood loss and acute phase pain.

## Abbreviations

DVT: Deep vein thrombosis
EBV: Estimated blood volume
Hb: Hemoglobin
Hct: Hematocrit
IPC: Intermittent pneumatic compression devices
PTE: Pulmonary thromboembolism
RBC: Red blood cell
TKA: Total knee arthroplasty
